# The Effectiveness of Artificial Intelligence-Based Interventions for Students with Learning Disabilities: A Systematic Review

**DOI:** 10.3390/brainsci15080806

**Published:** 2025-07-28

**Authors:** Andrea Paglialunga, Sergio Melogno

**Affiliations:** Department of Economic, Psychological, Communication, Education and Motor Sciences, Università Degli Studi Niccolò Cusano, 00166 Rome, Italy; sergio.melogno@unicusano.it

**Keywords:** artificial intelligence, learning disabilities, educational technology, personalized learning, assistive technology, systematic review, special education, dyslexia, cognitive support, adaptive learning systems

## Abstract

**Background/Objectives**: While artificial intelligence (AI) is rapidly transforming education, its specific effectiveness for students with learning disabilities (LD) requires rigorous evaluation. This systematic review aims to assess the efficacy of AI-based educational interventions for students with LD, with a specific focus on the methodological quality and risk of bias of the available evidence. **Methods**: A systematic search was conducted across seven major databases (Google Scholar, ScienceDirect, APA PsycInfo, ERIC, Scopus, PubMed) for experimental studies published between 2022 and 2025. This review followed PRISMA guidelines, using the PICOS framework for inclusion criteria. A formal risk of bias assessment was performed using the ROBINS-I and JBI critical appraisal tools. **Results**: Eleven studies (representing 10 independent experiments), encompassing 3033 participants, met the inclusion criteria. The most studied disabilities were dyslexia (six studies) and other specific learning disorders (three studies). Personalized/adaptive learning systems and game-based learning were the most common AI interventions. All 11 studies reported positive outcomes. However, the risk of bias assessment revealed significant methodological limitations: no studies were rated as having a low risk of bias, with most presenting a moderate (70%) to high/serious (30%) risk. Despite these limitations, quantitative results from the stronger studies showed large effect sizes, such as in arithmetic fluency (d = 1.63) and reading comprehension (d = −1.66). **Conclusions**: AI-based interventions demonstrate significant potential for supporting students with learning disabilities, with unanimously positive reported outcomes. However, this conclusion must be tempered by the considerable risk of bias and methodological weaknesses prevalent in the current literature. The limited and potentially biased evidence base warrants cautious interpretation. Future research must prioritize high-quality randomized controlled trials (RCTs) and longitudinal assessments to establish a definitive evidence base and investigate long-term effects, including the risk of cognitive offloading.

## 1. Introduction

Learning disabilities affect a significant portion of the student population, creating unique barriers to academic achievement that require specialized educational interventions. Students with conditions such as dyslexia, dyscalculia, and dysgraphia face distinct cognitive, academic, and behavioral challenges that traditional teaching approaches often struggle to address effectively. Despite the availability of established intervention methods, providing truly individualized support that adapts to each student’s specific learning patterns remains a persistent challenge in special education.

The emergence of artificial intelligence (AI) in education has opened unprecedented possibilities for addressing these challenges. Recent systematic reviews have documented AI’s transformative potential across educational settings, demonstrating significant positive impacts on academic performance, personalized learning, and educational management [1,2]. The democratization of sophisticated AI capabilities, particularly following the release of ChatGPT in November 2022, has accelerated the adoption of these technologies in educational contexts.

Current AI applications in education encompass machine learning tools, intelligent tutoring systems, chatbots, educational games, and virtual reality devices, showing promise in enhancing learning environments through adaptive feedback, automated evaluation, and real-time personalization [2,3]. However, implementation challenges persist, including digital divides, privacy concerns, insufficient teacher training, and questions about maintaining balanced human–technology integration [1,4].

Despite extensive documentation of AI’s general educational impact through multiple systematic reviews examining hundreds of studies [1,2], a critical gap exists in understanding AI applications specifically designed for students with learning disabilities. While previous reviews have examined AI’s broad educational influence, none have provided a comprehensive analysis of how these technologies address the specific needs of this vulnerable population.

AI-based interventions promise to revolutionize special education by offering scalable, customized solutions that adapt in real-time to individual learning patterns and challenges. From intelligent tutoring systems to game-based learning platforms, these technologies could provide the individualized support that students with learning disabilities need to reach their full potential. However, the specific effectiveness of these interventions for this population remains under-researched and lacks systematic evaluation.

This systematic review addresses this critical gap by focusing specifically on AI-based interventions for students with learning disabilities. The primary objectives are the following:

- RQ1: To what extent are AI-based educational interventions effective in improving learning outcomes for students with learning disabilities when compared to traditional teaching methods or control groups?
- RQ2: What specific types of AI technologies (e.g., personalized learning systems, generative AI, game-based learning, assistive apps) are most frequently studied, and which demonstrate the greatest effectiveness for students with learning disabilities?

By addressing these questions, this review seeks to provide educators, policymakers, and researchers with evidence-based insights to guide the implementation and further development of AI technologies in special education settings, ensuring that the transformative potential of AI extends to all learners, including those with learning disabilities.

## 2. Materials and Methods

This systematic review was conducted following the Preferred Reporting Items for Systematic Reviews and Meta-Analyses (PRISMA) 2020 guidelines [5]. To ensure comprehensive coverage and address the limitations of previous searches, a systematic search was performed across multiple electronic databases: Google Scholar, ScienceDirect, APA PsycInfo, ERIC, Scopus, and PubMed.

The search was conducted between April 2025 and July 2025 to identify experimental studies published from 2022 to the present. This timeframe was chosen to focus on the most recent advancements in AI, particularly following the widespread adoption of Large Language Models (LLMs).

The following search query was consistently applied across all databases, adapting to the different search formats:

“(“Large Language Models” OR “LLM” OR “Artificial Intelligence”) AND (“learning disabilities” OR “specific learning disorder”) AND (“students” OR “education” OR “academic support”)”

The search was limited to peer-reviewed articles published in English.

### 2.1. Inclusion and Exclusion Criteria (PICOS Framework)

To ensure a focused and rigorous review, inclusion and exclusion criteria were defined using the PICOS framework (Population, Intervention, Comparison, Outcomes, Study Design):

- Population (P): Studies involving students of any age (from primary school to university) with a formal diagnosis of a specific learning disability (SLD) as defined by established diagnostic criteria (e.g., DSM-5). This includes conditions such as dyslexia, dyscalculia, dysgraphia, and other specific learning disorders. Studies focusing primarily on intellectual disabilities or autism spectrum disorder (ASD) without a co-occurring SLD were excluded, though studies with mixed populations were considered if data for the SLD subgroup were reported;
- Intervention (I): The intervention had to be educational in nature and utilize an identifiable artificial intelligence (AI) component. This included personalized/adaptive learning systems, intelligent tutoring systems (ITS), AI-based games, generative AI applications, and AI-powered assistive technologies. Interventions where the AI component was not clearly described or was limited to basic automation were excluded;
- Comparison (C): Included studies were required to have a comparative design. This could involve a control group receiving traditional instruction or no intervention, a comparison with another form of technology, or a pre-test/post-test design where outcomes were compared against a baseline;
- Outcomes (O): Studies had to measure and report quantitative data on educational or cognitive outcomes. Primary outcomes of interest included academic performance (e.g., reading fluency, mathematical skills), cognitive functions (e.g., memory, attention), and student engagement. Studies that did not provide empirical data on learning outcomes were excluded;
- Study Design (S): Only peer-reviewed experimental or quasi-experimental studies were included. This encompasses randomized controlled trials (RCTs), non-randomized controlled trials, and single-subject designs. Literature reviews, meta-analyses, theoretical papers, conference abstracts, and dissertations were excluded.

Detailed compliance analysis for each study against these criteria is provided in Appendix A (Table A1, Table A2, Table A3, Table A4, Table A5, Table A6, Table A7, Table A8, Table A9, Table A10 and Table A11).

### 2.2. Study Selection and PRISMA Flow Diagram

**Figure 1:**
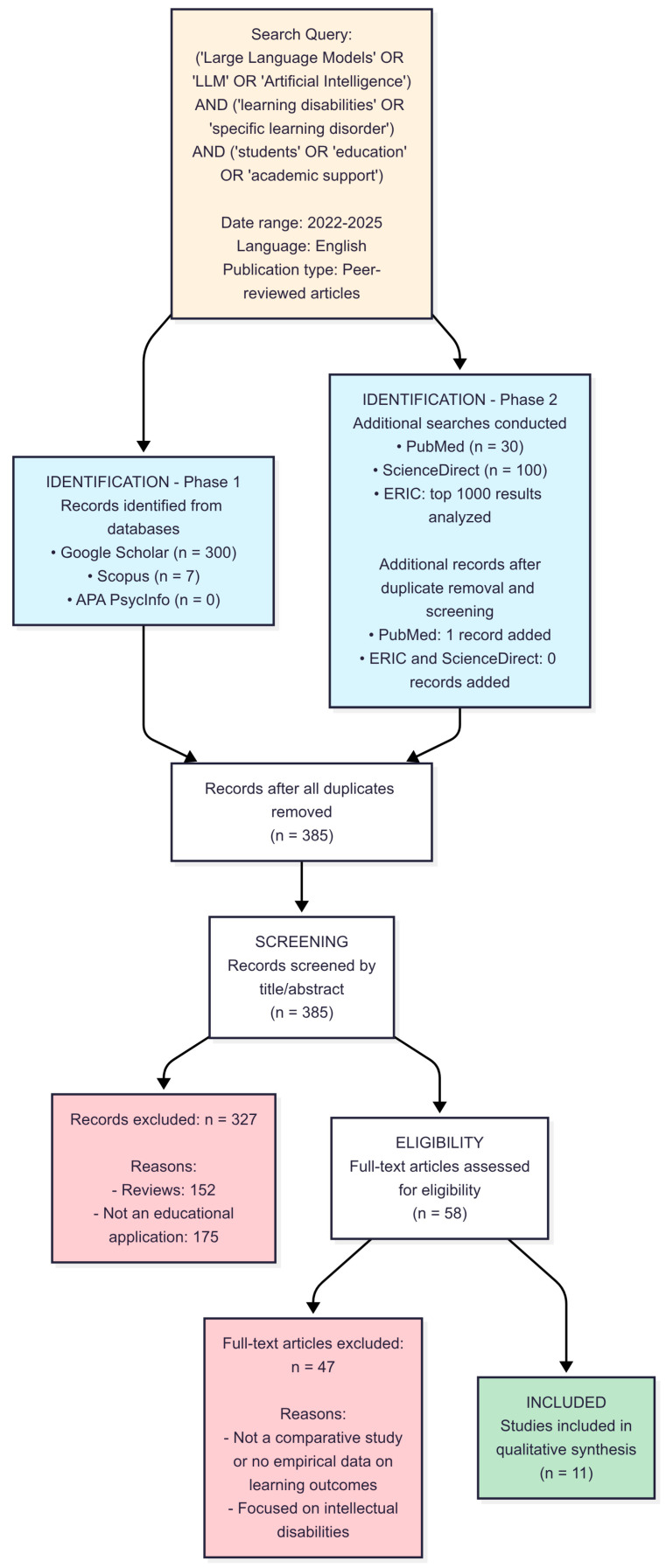
PRISMA diagram details.

The study selection process followed a systematic multi-stage screening procedure, as detailed in the PRISMA flow diagram (Figure 1). We independently conducted the full-text assessment to determine adherence to all inclusion criteria, with disagreements resolved through discussion and consensus. This rigorous evaluation process resulted in 11 studies meeting all criteria for inclusion in the final qualitative and quantitative syntheses.

### 2.3. Data Extraction

A standardized data extraction form was developed to systematically collect information from each of the 11 included studies. The extracted data were organized into three main categories to ensure comprehensive capture of relevant study characteristics and findings.

The first category encompassed general and methodological information, including the primary author and publication year, the specific learning disability under investigation, participant characteristics such as sample size and age or grade level, the study design employed, and details regarding the comparison group when applicable. This foundational information provided the necessary context for evaluating study quality and generalizability.

The second category focused on AI intervention details, capturing the type of AI technology utilized, such as personalized learning systems or generative AI applications, the specific name of the AI tool or platform when provided by the authors, and the context in which the intervention was implemented, whether in school, home, or clinical settings. This detailed characterization of interventions was essential for understanding the diversity of AI applications and their implementation contexts.

The third category addressed outcomes and effectiveness measures, documenting the specific learning outcomes assessed, such as reading comprehension or mathematical fluency, the measurement tools employed, including standardized tests and other assessment instruments, the reported effectiveness of interventions, and a comprehensive summary of key quantitative findings. This included effect sizes and *p*-values where available, providing the quantitative foundation necessary for evaluating intervention impact and facilitating potential future meta-analyses.

The authors independently extracted the data, and any discrepancies were resolved through discussion to ensure accuracy and consistency.

### 2.4. Risk of Bias Assessment

To address a key limitation of the original manuscript and to critically appraise the quality of the included studies, a formal risk of bias assessment was conducted. Given the heterogeneity of the study designs, two different validated tools were employed to ensure appropriate evaluation across the diverse methodological approaches represented in the literature.

For quasi-experimental studies, the Risk Of Bias In Non-randomised Studies of Interventions (ROBINS-I) tool was utilized, as this instrument provides a comprehensive framework for assessing bias across multiple domains in non-randomized intervention studies. For case series, case studies, and single-group pre–post studies, the appropriate JBI Critical Appraisal Checklist was applied, recognizing that these study designs require different methodological considerations and bias assessment criteria than comparative studies.

We independently assessed each study. The evaluation focused on domains such as confounding variables, selection of participants, classification of interventions, measurement of outcomes, and missing data. Based on the assessment, each study was assigned an overall risk of bias judgment (e.g., Low, Moderate, High, or Serious). A summary of this assessment is presented in the Results section, with more detailed evaluations provided in Appendix B (Table A12 and Table A13).

### 2.5. AI-Assisted Content Generation and Verification (GAMER Statement)

In adherence with the Guidelines for Reporting AI-Assisted Materials in Scholarly Work (GAMER) [6], this section provides a transparent account of how AI tools were utilized in the preparation of this manuscript.

Large Language Models (specifically, Claude 3 Opus and Google Gemini 2.5 pro) were used for the following tasks:

- Summarization: Assisting in the initial summarization of key findings manually extracted from the included articles;
- Data Organization: Helping to structure and organize the extracted data from the author notes into formatted tables for the Section 3 and Appendix A and Appendix B;
- Drafting Support: Aiding in the initial drafting of sections of the manuscript, particularly the abstract and parts of the discussion, by rephrasing and improving clarity.

The following step-wise human validation process was rigorously applied to all AI-generated content:

1. Source Verification: Every piece of information or data generated by the AI was manually cross-referenced with the original source articles and data files to ensure complete accuracy;
2. Fact-Checking: All claims, summaries, and quantitative data points were independently verified by the authors;
3. Critical Review and Editing: All AI-generated text was critically reviewed, rewritten, and edited by the authors to ensure it accurately reflected the source material, aligned with the study’s narrative, and met academic and scientific standards. The final manuscript represents the authors’ own work and intellectual contribution.

No AI tools were used for the core methodological tasks of this review, such as study selection, data extraction, or the risk of bias assessment, which were performed entirely by the human authors.

## 3. Results

The 11 included studies represent a diverse range of research conducted between 2022 and 2025. The studies varied significantly in terms of the specific learning disabilities addressed, participant demographics, AI technologies employed, and methodological design. For all subsequent percentage calculations and statistical analyses, we considered 10 independent experiments to avoid double-counting evidence from the overlapping studies.

It is important to note some methodological specificities regarding the selection and nature of the samples. For instance, while Samuelsson [7] did not use formal diagnostic criteria, the performance-based identification of math learning disabilities (MLD) through a bottom 25% cut-off on arithmetic fact fluency pre-tests represents a methodologically sound approach that aligns with established research practices in the field. This cut-off criterion is consistent with recommendations from multiple studies cited by Samuelsson, including Cowan & Powell [8], Geary et al. [9], and de Smedt & Gilmore [10], who have used similar percentile-based approaches to identify students with mathematical learning difficulties. Finally, in the case study by Rizos et al. [11], which included one participant with dyslexia and one with autism spectrum disorder, only the results pertaining to the participant with dyslexia were considered for this analysis, in line with the inclusion criteria.

The AI technologies were varied, with personalized/adaptive learning systems and game-based learning being the most common. Other interventions included assistive technologies (e.g., text-to-speech), recommendation systems, intelligent tutoring systems (ITS), and generative AI (ChatGPT).

Methodologically, the studies were predominantly quasi-experimental. The risk of bias assessment, detailed further in Section 3.1, revealed that no studies were rated as having a low risk of bias, with most presenting a moderate to high risk. A summary of the key characteristics of each included study is provided in Table 1.

### 3.1. Risk of Bias in Included Studies

The methodological quality of the 11 included studies was assessed using the ROBINS-I and JBI Critical Appraisal tools. The overall risk of bias was found to be considerable across the board. No studies were rated as having a “Low” risk of bias. The majority of studies (70%, *n* = 7) were assessed as having a “Moderate” risk of bias. Three studies (30%) were rated as “High” risk, and one study (10%) was rated as having a “Serious” risk of bias.

The most common methodological limitations identified were the lack of appropriate randomization, inadequate blinding of participants or outcome assessors, and the absence of a control group in several studies. Many studies were primarily focused on technological development, with less rigorous clinical evaluation. The detailed breakdown of the risk of bias for each study is presented in Table 1, with additional methodological details and study design information provided in Table 2.

### 3.2. Effectiveness of AI Interventions

All 11 studies reported positive outcomes, though methodological limitations require cautious interpretation. Key quantitative findings from stronger studies include a wide range of outcomes, from academic performance in reading and mathematics to cognitive functions like attention and memory. The interventions led to statistically significant improvements, often with large effect sizes. For example, Gharaibeh et al. [15] found that a ChatGPT-based intervention produced a very large effect on reading comprehension (Cohen’s d = −1.66) compared to traditional instruction. Similarly, Effect sizes across studies ranged from moderate to very large, with particularly strong outcomes observed for mathematics interventions and cognitive training programs (see Table 3 for detailed quantitative results).

Interventions using assistive technology also showed strong results. Chukwuemeka & Agbarakwe [19] found that the Speechify app led to significantly higher performance and retention in reading compared to two other teaching methods, with a mean gain of +15.20 points on their reading performance test. Cognitive training programs also yielded significant results; Fami et al. [20] reported a 77.53% mean improvement in working memory and a 40.37% improvement in academic function (reading) following their mixed cognitive intervention.

## 4. Discussion

This systematic review provides preliminary but compelling evidence that AI-based interventions can be effective in supporting students with learning disabilities. The unanimous positive outcomes reported across all 11 included studies, despite their methodological diversity, suggest a promising potential for these technologies. The interventions demonstrated success across a wide range of learning disabilities, including dyslexia and math disabilities, and were implemented in various contexts from primary schools to universities.

Moving beyond a simple declaration of effectiveness, the quantitative data reveals the magnitude of these positive impacts. For instance, an AI system for arithmetic practice yielded a large effect size (d = 0.80) for the general student population and an even larger effect (d = 1.63) for students with math learning disabilities [7]. These concrete figures underscore the potential of AI to deliver significant and measurable educational benefits.

### 4.1. Publication Bias and the Unanimity Problem

A critical concern that emerges from this systematic review is the potential for significant publication bias. The fact that all 11 included studies reported positive outcomes is statistically improbable and raises serious questions about the completeness of the available evidence base.

The unanimous positive results across diverse populations, interventions, and methodological approaches suggest systematic underreporting of negative or null findings. In any genuine research domain, some interventions would be expected to show no effect or even negative effects, especially given the methodological diversity of the included studies. This pattern is particularly concerning in AI and special education research, a nascent field where positive results may receive preferential treatment in publication decisions.

Unfortunately, the small number of included studies and their methodological heterogeneity preclude the construction of a funnel plot, which would have provided visual evidence of publication bias through asymmetrical distribution of effect sizes. This analytical limitation further compounds our inability to assess the true extent of missing negative or null results in the literature. Additionally, searches in grey literature sources yielded no relevant studies, suggesting that unpublished negative findings may be particularly difficult to access in this emerging research area.

### 4.2. Long-Term Effects and the Cognitive Offloading Paradox

A critical gap identified in this review is the near-complete absence of long-term follow-up. While studies report immediate gains, none have tracked students over extended periods to assess skill retention or potential negative consequences, such as “cognitive offloading”. This phenomenon, where learners become dependent on technology to perform tasks rather than developing the underlying cognitive skills, is a major concern, particularly for students with learning disabilities who need to strengthen, not bypass, their cognitive functions [21]. Empirical studies using neurophysiological measurements have begun to demonstrate that intensive use of AI assistants, while supportive, can reduce the user’s neural engagement, leading to the accumulation of a “cognitive debt” [22]. For instance, students using AI for math practice answered more problems correctly but scored lower on conceptual understanding tests, suggesting that AI may enhance procedural skills without fostering deeper learning [23]. To address this significant gap, future research must move beyond short-term efficacy studies and embrace comprehensive longitudinal designs that can capture the full spectrum of AI’s impact on learning processes.

We strongly recommend the adoption of longitudinal frameworks that incorporate specific follow-up periods at regular intervals, such as at 6, 12, and 24 months post-intervention, to systematically assess the durability of observed learning gains. These extended timeframes are essential for distinguishing between temporary performance improvements and sustained educational benefits that persist beyond the immediate intervention period.

Equally important is the systematic measurement of skill retention to determine whether improvements are maintained over time in the absence of the AI tool. This assessment is fundamental for understanding whether students develop genuine competency or become dependent on technological scaffolding for performance. Without such evaluation, we cannot determine if AI interventions produce lasting educational value or merely create performance illusions that dissipate when support is removed.

Research designs must also incorporate rigorous assessment of skill transfer, evaluating whether students can successfully apply learned skills to novel tasks and contexts that do not involve the AI intervention. This transfer assessment is particularly important for students with learning disabilities, who often struggle with generalizing skills across different contexts and may be especially vulnerable to developing narrow, context-dependent competencies.

Finally, longitudinal studies should include systematic monitoring for cognitive atrophy by periodically assessing core cognitive skills to ensure they are not degrading due to over-reliance on technology. This monitoring is essential for detecting any unintended consequences of AI dependency, such as the deterioration of fundamental cognitive processes that students might otherwise develop through more effortful, unassisted practice. Such comprehensive longitudinal approaches will provide the nuanced understanding necessary to guide responsible implementation of AI technologies in educational settings for students with learning disabilities.

### 4.3. Implications for Practice and Policy

For educators and practitioners, these findings suggest that AI tools can be powerful supplements to, but not replacements for, high-quality instruction. The key is to select the right tool for the right need, for example, using text-to-speech applications to support reading access while employing adaptive practice systems to build specific skills. AI tools can also serve as personalized support that is more “discreet” and less “embarrassing” than traditional disability services, reducing the stigma associated with seeking help [12]. A “complementary AI model,” where technology reduces extraneous cognitive load [24] while maintaining the necessary challenge for skill development, appears most promising.

For policymakers, the evidence supports the funding of pilot programs to explore the integration of AI in special education. However, given the methodological limitations of the current evidence base, widespread, mandated adoption would be premature. Policy should focus on creating frameworks for the rigorous evaluation of AI tools, ensuring they are not only effective but also equitable and safe. Furthermore, providing professional development for teachers is crucial for the successful and responsible implementation of these technologies.

### 4.4. Future Research Directions

Based on the findings and limitations of this review, several critical priorities emerge for advancing research in this field. Most urgently, there is a compelling need for well-designed randomized controlled trials with adequate sample sizes and active control groups to establish a more definitive evidence base. The current literature’s reliance on small-scale studies and quasi-experimental designs significantly limits our ability to draw robust conclusions about the efficacy of AI interventions for students with learning disabilities.

Longitudinal investigations represent another essential research priority, as detailed in Section 4.3. These studies are critical for understanding skill retention patterns and examining the potential for cognitive offloading effects that may emerge over extended periods of AI tool usage. Without such long-term perspectives, we cannot fully assess whether observed benefits represent genuine learning gains or temporary performance enhancements.

The research community must also confront the substantial risk of publication bias in this emerging field. Researchers should commit to pre-registering their studies and publishing all findings, including null or negative results, to create a more balanced and comprehensive evidence base. This transparency is particularly crucial given the commercial interests and technological enthusiasm that may influence publication patterns in AI research.

Moving forward, research should evolve beyond simply asking whether AI is effective to investigating more nuanced questions about differential effectiveness. Specifically, studies should examine which AI technologies demonstrate the greatest efficacy, for which specific learning disabilities, and under which particular instructional conditions. This granular approach will provide the detailed guidance necessary for evidence-based implementation decisions.

Student voice represents a notably underexplored dimension in current research. In-depth qualitative assessments of student perceptions are essential for understanding how AI influences the learning experience and for informing responsible implementation strategies. Students with learning disabilities possess unique insights into how these tools can support self-advocacy and learning autonomy, perspectives that are crucial for developing truly supportive interventions.

The field would benefit substantially from developing and adopting a core set of standardized outcome measures to facilitate meaningful comparison and synthesis across studies. The current heterogeneity in assessment approaches severely limits our ability to build cumulative knowledge and conduct robust meta-analyses.

Finally, given the extensive body of research examining AI applications in general education populations, future studies must prioritize disaggregated data reporting when samples include students with learning disabilities. Many existing studies that evaluate AI interventions in mixed populations fail to provide separate analyses for students with learning disabilities, thereby limiting our understanding of differential effects and potentially masking benefits or risks specific to this vulnerable population. We strongly recommend that researchers conducting AI studies in educational settings explicitly report outcomes separately for students with and without learning disabilities when both populations are included in their samples, as this disaggregated approach is essential for building an evidence base that truly serves the needs of students with learning disabilities.

### 4.5. Conclusions

This systematic review indicates that AI-based interventions hold significant, tangible promise for improving academic and cognitive outcomes for students with learning disabilities. The evidence, though preliminary and methodologically limited, is unanimously positive and shows that AI can provide powerful, personalized support. However, the path forward requires a balanced and critical approach. Educators, policymakers, and researchers must work together to foster innovation while demanding rigorous evidence of long-term effectiveness and safety. By focusing on high-quality research and responsible implementation, the field can harness the transformative potential of AI to create more equitable and effective learning environments for all students.

## Figures and Tables

**Table 1:** Characteristics of Included Studies (Part 1/2).

Author and Year	Disability Studied	Sample and Age	AI Technology and Tools
Zingoni et al. (2024) [12]	Dyslexia	50 university students	Personalized Sys., VR (VRAIlexia)
Ayasrah et al. (2024) [13]	SLD	15 students (primary)	Assistive Tech, Games (PROKET)
Morciano et al. (2024) [14]	Dyslexia	1237 students (main) + 50 (validation)	Recommendation System
Gharaibeh et al. (2025) [15]	Dyslexia	60 children (8–11 y)	Gen-AI (ChatGPT)
Wang et al. (2022) [16]	Dyslexia	20 student datasets	AI-A^2^C System
Hany et al. (2024) [17]	Dyslexia	392 individuals (9–11 y)	ITS, Games (Nexia Tutor)
Sukasih et al. (2024) [18]	SLD	40 students (Gr 4–6)	Game-Based Learning
Rizos et al. (2024) [11]	Dyslexia, ASD	2 students (8th gr)	Gen-AI (ChatGPT)
Chukwuemeka & Agbarakwe (2024) [19]	Dyslexia	205 students (S.S.1)	Assistive Tech (Speechify)
Samuelsson (2023) [7]	Math Disabilities	1006 students (8 y)	Personalized System
Fami et al. (2024) [20]	SLD (primarily dyslexia)	6 children (10–13 y)	Mixed Cognitive Program (Mobin)

**Table 2:** Characteristics of Included Studies (Part 2/2).

Author and Year	Study Design	Overall Risk of Bias	Methodological Notes
Zingoni et al. (2024) [12]	Descriptive/Case series	Moderate	Same population as Morciano et al. (2024) [14].
Ayasrah et al. (2024) [13]	Pre-post (single group)	High	-
Morciano et al. (2024) [14]	Algorithm development with quasi-experimental validation	Moderate	Same population as Zingoni et al. (2024) [12].
Gharaibeh et al. (2025) [15]	Quasi-experimental	Moderate	-
Wang et al. (2022) [16]	Quasi-experimental	Serious	-
Hany et al. (2024) [17]	Descriptive/Case series	High	-
Sukasih et al. (2024) [18]	Quasi-experimental (pre-post)	High	-
Rizos et al. (2024) [11]	Case study	Moderate	Only data from the participant with dyslexia were analyzed.
Chukwuemeka & Agbarakwe (2024) [19]	Quasi-experimental	Moderate	-
Samuelsson (2023) [7]	Quasi-experimental	Moderate	MLD population defined by performance (bottom 25% cut-off), not diagnosis.
Fami et al. (2024) [20]	Single-subject (A-B-A)	Moderate	-

**Table 3:** Quantitative Results Summary Table.

Author (Year)	Brief Quantitative/Qualitative Summary
Zingoni et al. (2024) [12]; Morciano et al. (2024) [14]	A hybrid recommendation system (MAE = 0.8093) improved academic scores for dyslexic students by +1.1 points (15.5% improvement) in a validation study (n = 50).
Ayasrah et al. (2024) [13]	The PROKET technology program led to significant reductions in attention (10.18%) and memory (9.33%) difficulties for students with SLD (n = 15), with large effect sizes (Hedges’ g > 1.5).
Gharaibeh & Basulayyim (2025) [15]	A ChatGPT-based intervention for Arabic-speaking children with dyslexia (n = 60) resulted in a significant improvement in reading comprehension compared to a control group (*p* < 0.001), with a large effect size (Cohen’s d = 1.66).
Wang et al. (2022) [16]	The AI-A^2^C system achieved high accuracy (94.98–98.57%) and efficiency (96.95–99.54%) on classification tasks for dyslexia support (n = 20 datasets). Compared to baseline AI classifiers, it reduced user effort by 36.56% and interaction time by 66.34%.
Hany et al. (2024) [17]	The AI tutoring service led to significant but unquantified improvements in phonological awareness and visual memory for dyslexic students (n = 392).
Sukasih et al. (2024) [18]	An AI-based games intervention for students with SLD (n = 40) improved overall literacy skills by 39% and learning motivation by 42%. Digital literacy saw the largest gain at 56%.
Rizos et al. (2024) [11]	In a case study (n = 2), ChatGPT-generated worksheets led to notable but unquantified improvements in test performance for the student with dyslexia. The main quantitative result was high self-reported satisfaction (5/5 on a Likert scale).
Chukwuemeka & Agbarakwe (2024) [19]	The Speechify AI app led to a mean gain of +15.20 points in reading performance for dyslexic students (n = 205), significantly outperforming collaborative learning (+10.21 points) and discussion methods (+8.26 points).
Samuelsson (2023) [7]	For the general student population (n = 1006), an AI intervention was most effective for arithmetic fluency (d = 0.80). For students with math difficulties (n = 246), memorization was most effective (d = 1.94), though AI was also highly effective (d = 1.63).
Fami et al. (2024) [20]	A mixed cognitive intervention for children with SLD (n = 6) showed significant improvements in working memory (+77.53%), processing speed (+47.19%), attention (+51.44%), and reading skills (+40.37%).

## Data Availability

No new data were created or analyzed in this study.

## Abbreviations

The following abbreviations are used in this manuscript:

AI	Artificial Intelligence
ASD	Autism Spectrum Disorder
AI-A^2^C	AI-based Augmentative Alternative Communication
ChatGPT	Chat Generative Pre-trained Transformer
DSQ	Dyslexia Screening Questionnaire
GAMER	Guidelines for Reporting AI-Assisted Materials in Scholarly Work
ITS	Intelligent Tutoring Systems
JBI	Joanna Briggs Institute
LD	Learning Disabilities
LLM	Large Language Models
MLD	Math Learning Disabilities
PICOS	Population, Intervention, Comparison, Outcomes, Study Design
PRISMA	Preferred Reporting Items for Systematic Reviews and Meta-Analyses
RCT	Randomized Controlled Trial
ROBINS-I	Risk Of Bias In Non-randomised Studies of Interventions
SLD	Specific Learning Disorder(s)
VR	Virtual Reality

## Appendix A. Detailed PICOS Analysis per Study

**Table A1:** Detailed PICOS Analysis per Study—Population (P) Summary Table (Part 1/3).

Study	Participant Age	Diagnosis
Zingoni et al. (2024) [12]	University students (≥18 years) and up to 5 years after leaving university	Dyslexia (specific learning disorder)
Ayasrah et al. (2024) [13]	Primary school students (typically 6–12 years)	Specific learning disorders (SLD)—formally diagnosed
Morciano et al. (2024) [14]	University students (≥18 years) and up to 5 years after leaving university	Dyslexia (specific learning disorder)—valid diagnosis required
Gharaibeh et al. (2025) [15]	8–11 years	Dyslexia (specific learning disorder with impairment in reading) according to DSM-5 criteria
Wang et al. (2022) [16]	School and college students (implies 5–25 years)	Dyslexia
Hany et al. (2024) [17]	9–11 age group (within broader 7–17 range)	Dyslexia
Sukasih et al. (2024) [18]	Elementary school students grades 4–6 (9–12 years)	Specific learning disorders (SLD), including dyslexia, dyscalculia, and dysgraphia
Rizos et al. (2024) [11]	14 years old (8th grade)	One student with dyslexia, one with autism spectrum disorder (ASD)
Chukwuemeka & Agbarakwe (2024) [19]	Senior secondary school 1 (approx. 15–16 years old)	Dyslexic students identified via Dyslexia Screening Questionnaire (DSQ)
Samuelsson (2023) [7]	8-year-old students (Year 2)	Mathematics learning disabilities (MLD)—lowest 25% on pre-test
Fami et al. (2024) [20]	10–13 years	Specific learning disorder (SLD), primarily dyslexia

**Table A2:** Detailed PICOS Analysis per Study—Population (P) Summary Table (Part 2/3).

Study	Sample Size	Setting
Zingoni et al. (2024) [12]	n = 1237 (main questionnaire), n = 50 (validation test), n = 100+ (VR tests)	Italian universities (66 universities involved)
Ayasrah et al. (2024) [13]	n = 15 (8 males, 7 females)	Education Challenges Center in Amman, Jordan, (formal educational setting)
Morciano et al. (2024) [14]	n = 1237 (main dataset), n = 50 (validation study: 40% dyslexic, 60% non-dyslexic)	Italian universities (native Italian speakers)
Gharaibeh et al. (2025) [15]	n = 60 (30 experimental group, 30 control group)	Public and private schools in the United Arab Emirates (UAE)
Wang et al. (2022) [16]	n = 20 dyslexia students datasets	Educational environment (schools and colleges)
Hany et al. (2024) [17]	n = 392 dyslexic participants, n = 3252 non-dyslexic (total n = 3644)	Recruited through dyslexia associations and centers
Sukasih et al. (2024) [18]	n = 40 elementary school students with SLD	Three inclusive elementary schools in Semarang City, Indonesia
Rizos et al. (2024) [11]	n = 2 students with special educational needs	General mathematics classroom in Greece
Chukwuemeka & Agbarakwe (2024) [19]	n = 205 dyslexic students	Public secondary schools in Port Harcourt Metropolis, Nigeria
Samuelsson (2023) [7]	n = 1006 total students; MLD subset n = 246	Primary school mathematics education in Sweden
Fami et al. (2024) [20]	n = 6	Primary schools (grades 3–6)

**Table A3:** Detailed PICOS Analysis per Study—Population (P) Summary Table (Part 3/3).

Study	Compliance Analysis	Notes/Deviations
Zingoni et al. (2024) [12]	- Specific learning disability diagnosis—Dyslexia clearly identified - Formal educational setting—University context - Absence of exclusions (ID, primary ASD, sensory impairments)—Not mentioned as exclusion criteria	None. This study focuses on university students with dyslexia in formal educational settings.
Ayasrah et al. (2024) [13]	- Specific learning disability diagnosis—Formally diagnosed SLD according to DSM-5 criteria - Formal educational setting—Education Challenges Center (specialized educational institution) - Absence of exclusions (ID, primary ASD, sensory impairments)—Not mentioned as exclusion criteria	None. The population clearly meets all inclusion criteria with formally diagnosed SLD students in an appropriate educational setting.
Morciano et al. (2024) [14]	- Specific learning disability diagnosis—Valid dyslexia diagnosis required - Formal educational setting—University context - Absence of exclusions (ID, primary ASD, sensory impairments)—Not mentioned as exclusion criteria	None. This study focuses on university students with dyslexia in formal educational settings.
Gharaibeh et al. (2025) [15]	- Specific learning disability diagnosis—Dyslexia clearly diagnosed - Formal educational setting—Public and private schools - Absence of exclusions—IQ 90–120 (average-high), no intellectual disability, sensory, or primary ASD	No significant deviations. The population is perfectly aligned with the inclusion criteria.
Wang et al. (2022) [16]	- Specific learning disability diagnosis—Dyslexia clearly identified as target population - Formal educational setting—School and college context explicitly mentioned - Absence of exclusions (ID, primary ASD, sensory impairments)—Focus specifically on dyslexia	This study clearly targets dyslexic students in educational settings. While demographic details are limited, the population meets inclusion criteria.
Hany et al. (2024) [17]	- Specific learning disability diagnosis—Dyslexia clearly identified and diagnosed - Formal educational setting—Recruited through dyslexia centers rather than schools directly - Absence of exclusions (ID, primary ASD, sensory impairments)—Not mentioned as exclusion criteria	While this study includes diagnosed dyslexic participants, recruitment was through dyslexia associations/centers rather than formal educational settings. The large sample size strengthens the population validity.
Sukasih et al. (2024) [18]	- Specific learning disability diagnosis—SLD (dyslexia, dyscalculia, dysgraphia) professionally diagnosed - Formal educational setting—Inclusive elementary schools - Absence of exclusions (ID, primary ASD, sensory impairments)—Not mentioned as exclusion criteria	None. The population clearly meets all inclusion criteria with professionally diagnosed SLD students in appropriate educational setting.
Rizos et al. (2024) [11]	- Specific learning disability diagnosis—Dyslexia clearly identified (one student) - Formal educational setting—General mathematics classroom - Exclusion criteria—Includes one student with ASD as primary diagnosis	This study includes one student with ASD as a primary diagnosis, which is listed as an exclusion criterion. However, one student has dyslexia, making this a mixed compliance situation.
Chukwuemeka & Agbarakwe (2024) [19]	- Specific learning disability diagnosis—Dyslexic students identified through validated screening questionnaire - Formal educational setting—Public secondary school environment - Absence of exclusions (ID, primary ASD, sensory impairments)—Not mentioned as exclusion criteria	None. The population clearly meets all inclusion criteria with dyslexic students identified through systematic screening in formal educational settings.
Samuelsson (2023) [7]	- Specific learning disability diagnosis—MLD students identified through performance-based criteria (bottom 25%) - Formal educational setting—Primary school mathematics classes - Absence of exclusions (ID, primary ASD, sensory impairments)—Not mentioned as exclusion criteria	This study includes both general population and MLD students, with MLD defined functionally rather than through formal diagnosis. This approach is acceptable.
Fami et al. (2024) [20]	- Specific learning disability diagnosis—SLD according to DSM-5 criteria - Formal educational setting—students enrolled in grades 3–6 - Absence of exclusions (ID, primary ASD, sensory impairments)—appropriate exclusion criteria	Very small sample size (n = 6) for a pilot study. Diagnosis is generically defined as “SLD, primarily dyslexia” without detailed specification of subtypes.

**Table A4:** Detailed PICOS Analysis per Study—Intervention (I) Summary Table (Part 1/3).

Study	AI Type	Description
Zingoni et al. (2024) [12]	Machine Learning algorithms (Random Forest, SVM, etc.), Recommendation Systems, Neural Networks	VRAIlexia framework combining AI and VR to provide personalized learning strategies and study tools for dyslexic students
Ayasrah et al. (2024) [13]	PROKET Technology Program, based on OSMO principles. Uses AI to interpret children’s responses and gestures.	An interactive digital learning program that combines physical action with AI. It includes 12 games on an iPad for attention and memory.
Morciano et al. (2024) [14]	Recommendation Systems (collaborative filtering), Machine Learning algorithms	AI-powered recommendation system to suggest personalized learning tools and study strategies for dyslexic students
Gharaibeh et al. (2025) [15]	ChatGPT (GPT-3 and GPT-4 models) for personalized reading instruction	Interactive reading sessions with ChatGPT, personalized comprehension exercises, immediate feedback, phonological support
Wang et al. (2022) [16]	AI-based Augmentative Alternative Communication (AI-A^2^C) system with hybrid AI classifier	AI-powered communication system using pictograms and classification algorithms to reduce interaction effort and time
Hany et al. (2024) [17]	Multi-component AI system (Random Forest, BERT, DALL-E, Google Voice)	Comprehensive AI system with dyslexia screening, personalized reports, and game-based tutoring
Sukasih et al. (2024) [18]	Games-based Artificial Intelligence with adaptive learning capabilities	AI-integrated educational games providing adaptive materials, accommodating various learning styles, and adjusting difficulty
Rizos et al. (2024) [11]	ChatGPT 3.5 for generating personalized math worksheets and lesson plans	AI-generated worksheets tailored to students’ special educational needs and interests (square roots, irrational numbers)
Chukwuemeka & Agbarakwe (2024) [19]	Speechify App—AI-powered text-to-speech application	Text-to-speech technology that reads various electronic documents with highlighting and natural voice reading
Samuelsson (2023) [7]	AI engine for personalized arithmetic fact practice	Computer-based AI system that learns student’s knowledge needs to provide targeted number combination practice
Fami et al. (2024) [20]	“Mobin” program—web-based system with computer-based and home-based components	24 computerized cognitive exercises + home-based card activities, targeting executive functions

**Table A5:** Detailed PICOS Analysis per Study—Intervention (I) Summary Table (Part 2/3).

Study	Duration	Setting
Zingoni et al. (2024) [12]	Multiple components—questionnaire, VR test sessions, validation study	University/research environment
Ayasrah et al. (2024) [13]	12 sessions, each lasting 35–45 min.	Education Challenges Center.
Morciano et al. (2024) [14]	Single intervention with validation study	University/research environment
Gharaibeh et al. (2025) [15]	Multiple sessions during regular school hours	School classrooms
Wang et al. (2022) [16]	Evaluation period with pre-post assessment	Educational environment with AI-based assistive technology
Hany et al. (2024) [17]	Ongoing personalized tutoring with pre- and post-assessments	Remote accessibility
Sukasih et al. (2024) [18]	8 weeks of intervention	Inclusive elementary schools
Rizos et al. (2024) [11]	9 h of teaching intervention over multiple sessions	General mathematics classroom
Chukwuemeka & Agbarakwe (2024) [19]	3-week intervention period	Secondary school classrooms
Samuelsson (2023) [7]	6 weeks, 10 min per mathematics lesson	Primary school classroom environment
Fami et al. (2024) [20]	10 sessions (2 sessions/week for 5 weeks)	Clinical center + home

**Table A6:** **Appendix B**: Detailed PICOS Analysis per Study - Intervention (I) Summary Table (Part 3/3).

Study	Compliance Analysis	Notes/Deviations
Zingoni et al. (2024) [12]	- AI-based intervention—Multiple AI technologies clearly described - Educational purpose—Personalized learning support - Minimum duration respected—Multiple sessions - Appropriate setting—Educational/research context	None. The intervention clearly meets all AI-based educational intervention criteria.
Ayasrah et al. (2024) [13]	- AI-based intervention—Uses AI to interpret user interactions - Educational purpose—Focused on improving developmental skills - Minimum duration respected—Multiple sessions - Appropriate setting—Educational/clinical environment	The program’s use of AI to interpret actions and responses qualifies it as an AI-based intervention.
Morciano et al. (2024) [14]	- AI-based intervention—Clear use of machine learning recommendation systems - Educational purpose—Personalized learning support - Minimum duration respected—Single session with validation - Appropriate setting—Educational/research context	None. The intervention clearly meets all AI-based educational intervention criteria with sophisticated ML algorithms.
Gharaibeh et al. (2025) [15]	- AI-based intervention—ChatGPT clearly identified as generative AI - Educational purpose—Improving reading comprehension - Minimum duration respected—Multiple sessions - Appropriate setting—Formal educational environment	None. The intervention represents an appropriate educational use of AI.
Wang et al. (2022) [16]	- AI-based intervention—Clear hybrid AI classifier system - Educational purpose—Improving academic skills and communication - Minimum duration respected—Evaluation includes temporal assessment - Appropriate setting—Educational technology implementation	The intervention clearly meets all AI-based educational intervention criteria with a sophisticated multi-algorithm approach.
Hany et al. (2024) [17]	- AI-based intervention—Multiple AI technologies integrated - Educational purpose—Personalized language learning - Minimum duration respected—Ongoing tutoring - Appropriate setting—Educational technology platform	None. The intervention clearly meets all AI-based educational intervention criteria with comprehensive technology integration.
Sukasih et al. (2024) [18]	- AI-based intervention—Games-based AI with adaptive learning described - Educational purpose—Differentiated learning and literacy skills - Minimum duration respected—8 weeks - Appropriate setting—Inclusive schools	None. The intervention clearly meets all AI-based educational intervention criteria.
Rizos et al. (2024) [11]	- AI-based intervention—ChatGPT 3.5 clearly described - Educational purpose—Personalized math materials - Minimum duration respected—9 hours - Appropriate setting—Classroom environment	None. The intervention clearly meets all AI-based educational intervention criteria.
Chukwuemeka & Agbarakwe (2024) [19]	- AI-based intervention—Speechify incorporates AI for text-to-speech - Educational purpose—Reading comprehension and retention - Minimum duration respected—3-week period - Appropriate setting—School-based environment	None. The intervention clearly meets all AI-based educational intervention criteria with an assistive technology focus.
Samuelsson (2023) [7]	- AI-based intervention—Clear AI engine with adaptive learning - Educational purpose—Arithmetic fact fluency development - Minimum duration respected—6-week period - Appropriate setting—School-based mathematics education	None. The intervention clearly meets all AI-based educational intervention criteria with personalized adaptive learning.
Fami et al. (2024) [20]	- AI-based intervention—Web-based system with adaptive elements but not clearly defined as AI - Educational purpose—Improving cognitive and academic functions - Minimum duration met—10 sessions - Appropriate setting—Center + home	The intervention is described as "computer-based" but it is not clearly specified whether it uses artificial intelligence. It appears to be more of a traditional computerized program with adaptive elements.

**Table A7:** Detailed PICOS Analysis per Study—Comparator (C) Summary Table (Part 1/2).

Study	Comparator Details
Zingoni et al. (2024) [12]	- Control type: Random recommendations vs. AI-generated personalized recommendations - Comparison: Students receiving AI-based suggestions vs. students receiving random recommendations
Ayasrah et al. (2024) [13]	- Control type: Pre-post single group design. - Comparison: Pre-intervention measurements are compared with post-intervention measurements within the same group.
Morciano et al. (2024) [14]	- Control type: Students receiving AI-generated personalized recommendations vs. students receiving random recommendations - Comparison: Validation study comparing AI recommendations vs. random recommendations in 50 students
Gharaibeh et al. (2025) [15]	- Control type: Control group with standard traditional instruction according to Ministry of Education (MoE) criteria - Comparison: Experimental group (ChatGPT + standard instruction) vs. control group (standard instruction only)
Wang et al. (2022) [16]	- Control type: Comparison with existing AI classifiers (ME, SVM, CNB, NB) as baseline conditions - Comparison: Performance comparison between proposed hybrid AI-A^2^C system and individual AI algorithms
Hany et al. (2024) [17]	- Control type: Pre-post assessment design - Comparison: This study evaluated the effectiveness of the tutoring service through pre- and post-assessments of the participants.
Sukasih et al. (2024) [18]	- Control type: Quasi-experimental design with pre-test and post-test measurements - Comparison: Pre-intervention vs. post-intervention measurements within the same group
Rizos et al. (2024) [11]	- Control type: Comparison between AI-generated personalized worksheets vs. standard worksheets for other students - Comparison: Two students with special needs received AI-generated worksheets while 23 other students received standard worksheets
Chukwuemeka & Agbarakwe (2024) [19]	- Control types: Two comparison groups: 1. Collaborative Learning group, 2. Discussion Method group - Comparison: Speechify App (AI intervention) vs. Collaborative Learning vs. Discussion Method
Samuelsson (2023) [7]	- Control types: Three comparison groups: 1. Memorization (Mem), 2. Guided Learning (GL), 3. Control group (standard teaching) - Comparison: AI group compared against all three control conditions
Fami et al. (2024) [20]	- Control type: A-B-A design (single-subject research design) - Comparison: Pre-test vs. post-test with baseline measurements

**Table A8:** Detailed PICOS Analysis per Study—Comparator (C) Summary Table (Part 2/2).

Study	Compliance Analysis	Notes/Deviations
Zingoni et al. (2024) [12]	- Appropriate comparator—Yes, random recommendations provide a valid baseline. - No AI in control—Yes, the control group received non-AI-based (random) recommendations.	None. The comparator is appropriate for evaluating the effectiveness of the AI-based personalization.
Ayasrah et al. (2024) [13]	- Appropriate comparator—No, this is a pre-post single group design without a separate control group. - No AI in control—N/A.	This study lacks a control group, which limits the ability to attribute outcomes solely to the intervention.
Morciano et al. (2024) [14]	- Appropriate comparator—Yes, random recommendations provide a valid baseline. - No AI in control—Yes, the control group received non-AI-based (random) recommendations.	None. The comparator is appropriate for evaluating the effectiveness of the AI-based personalization.
Gharaibeh et al. (2025) [15]	- Appropriate comparator—Yes, standard instruction is a valid control. - No AI in control—Yes, the control group received traditional instruction without AI.	None. The comparator is appropriate and well-defined.
Wang et al. (2022) [16]	- Appropriate comparator—Yes, comparison with existing AI classifiers is a valid approach. - No AI in control—No, the comparison is between different AI models.	This study compares different AI models rather than AI vs. no-AI, which is a deviation from the PICOS criteria.
Hany et al. (2024) [17]	- Appropriate comparator—No, this is a pre-post assessment design without a separate control group. - No AI in control—N/A.	This study lacks a control group, which limits the ability to attribute outcomes solely to the intervention.
Sukasih et al. (2024) [18]	- Appropriate comparator—No, this is a pre-post single group design without a separate control group. - No AI in control—N/A.	This study lacks a control group, which limits the ability to attribute outcomes solely to the intervention.
Rizos et al. (2024) [11]	- Appropriate comparator—Yes, standard worksheets provide a valid baseline. - No AI in control—Yes, the control group received standard non-AI worksheets.	None. The comparator is appropriate for evaluating the effectiveness of the AI-based personalization.
Chukwuemeka & Agbarakwe (2024) [19]	- Appropriate comparator—Yes, both collaborative learning and discussion methods are valid active controls. - No AI in control—Yes, both control groups used non-AI teaching methods.	None. The comparators are appropriate and well-defined.
Samuelsson (2023) [7]	- Appropriate comparator—Yes, memorization, guided learning, and standard teaching are all valid controls. - No AI in control—Yes, all control groups used non-AI teaching methods.	None. The comparators are appropriate and well-defined.
Fami et al. (2024) [20]	- Appropriate comparator—No, this is a single-subject A-B-A design without a separate control group. - No AI in control—N/A.	This study lacks a control group, which limits the ability to attribute outcomes solely to the intervention.

**Table A9:** Detailed PICOS Analysis per Study—Outcomes (O) Summary Table (Part 1/2).

Study	Primary Outcomes	Secondary Outcomes
Zingoni et al. (2024) [12]	- Identification of learning strategies and tools for dyslexic students - Validation of AI-based recommendations	- User satisfaction with the VRAIlexia framework
Ayasrah et al. (2024) [13]	- Improvement in attention and memory skills (measured by Wechsler Intelligence Scale for Children)	- Enhancement of developmental skills
Morciano et al. (2024) [14]	- Accuracy of AI in recommending personalized learning tools - Validation of the recommendation system	- User satisfaction with the recommended tools
Gharaibeh et al. (2025) [15]	- Improvement in reading comprehension skills (measured by pre-post tests)	- Student engagement and motivation
Wang et al. (2022) [16]	- Reduction in interaction effort and time for students with dyslexia - Accuracy of the AI-A^2^C system	- User satisfaction with the communication system
Hany et al. (2024) [17]	- Improvement in language skills (reading, writing, phonological awareness) - Accuracy of the dyslexia screening tool	- User engagement with the game-based tutoring
Sukasih et al. (2024) [18]	- Improvement in literacy skills (reading and writing) - Effectiveness of differentiated learning	- Student motivation and engagement
Rizos et al. (2024) [11]	- Improvement in mathematics performance (square roots and irrational numbers)	- Student engagement and interest in mathematics
Chukwuemeka & Agbarakwe (2024) [19]	- Improvement in reading comprehension and retention	- Student performance compared across different teaching methods
Samuelsson (2023) [7]	- Improvement in arithmetic fact fluency	- Comparison of learning gains across different instructional methods
Fami et al. (2024) [20]	- Improvement in executive functions (e.g., working memory, planning) - Enhancement of academic functions	- Feasibility of the “Mobin” program

**Table A10:** Detailed PICOS Analysis per Study—Outcomes (O) Summary Table (Part 2/2).

Study	Compliance Analysis	Notes/Deviations
Zingoni et al. (2024) [12]	- Relevant educational outcomes—Yes, focuses on learning strategies and tools. - Measurable outcomes—Yes, validated through testing.	None. The outcomes are relevant and measurable.
Ayasrah et al. (2024) [13]	- Relevant educational outcomes—Yes, attention and memory are crucial for learning. - Measurable outcomes—Yes, measured by a standardized test.	None. The outcomes are relevant and measurable.
Morciano et al. (2024) [14]	- Relevant educational outcomes—Yes, focuses on personalized learning tools. - Measurable outcomes—Yes, validated through testing.	None. The outcomes are relevant and measurable.
Gharaibeh et al. (2025) [15]	- Relevant educational outcomes—Yes, reading comprehension is a key academic skill. - Measurable outcomes—Yes, measured by pre-post tests.	None. The outcomes are relevant and measurable.
Wang et al. (2022) [16]	- Relevant educational outcomes—Yes, focuses on communication and interaction efficiency. - Measurable outcomes—Yes, measured by time and accuracy metrics.	None. The outcomes are relevant and measurable.
Hany et al. (2024) [17]	- Relevant educational outcomes—Yes, focuses on core language skills. - Measurable outcomes—Yes, assessed through pre-post testing.	None. The outcomes are relevant and measurable.
Sukasih et al. (2024) [18]	- Relevant educational outcomes—Yes, focuses on literacy skills. - Measurable outcomes—Yes, measured by pre-post tests.	None. The outcomes are relevant and measurable.
Rizos et al. (2024) [11]	- Relevant educational outcomes—Yes, focuses on mathematics performance. - Measurable outcomes—Yes, assessed through worksheets and tests.	None. The outcomes are relevant and measurable.
Chukwuemeka & Agbarakwe (2024) [19]	- Relevant educational outcomes—Yes, reading comprehension and retention are key academic skills. - Measurable outcomes—Yes, measured by standardized tests.	None. The outcomes are relevant and measurable.
Samuelsson (2023) [7]	- Relevant educational outcomes—Yes, arithmetic fluency is a key academic skill. - Measurable outcomes—Yes, measured by standardized tests.	None. The outcomes are relevant and measurable.
Fami et al. (2024) [20]	- Relevant educational outcomes—Yes, executive and academic functions are crucial for learning. - Measurable outcomes—Yes, assessed through standardized tests.	None. The outcomes are relevant and measurable.

**Table A11:** Detailed PICOS Analysis per Study—Study Design (S) Summary Table.

Study	Study Design	Compliance Analysis	Notes/Deviations
Zingoni et al. (2024) [12]	Validation study with a between-subjects design (AI vs. random recommendations)	- Experimental or quasi-experimental design—Yes, it is a validation study. - Minimum duration—Yes, multiple sessions.	None. The study design is appropriate for the research question.
Ayasrah et al. (2024) [13]	Pre-post single group design	- Experimental or quasi-experimental design—Yes, it is a pre-post design. - Minimum duration—Yes, 12 sessions.	The lack of a control group is a limitation of this study design.
Morciano et al. (2024) [14]	Validation study with a between-subjects design (AI vs. random recommendations)	- Experimental or quasi-experimental design—Yes, it is a validation study. - Minimum duration—Yes, single session with validation.	None. The study design is appropriate for the research question.
Gharaibeh et al. (2025) [15]	Randomized Controlled Trial (RCT)	- Experimental or quasi-experimental design—Yes, it is an RCT. - Minimum duration—Yes, multiple sessions.	None. The study design is appropriate and robust.
Wang et al. (2022) [16]	Comparative study of different AI classifiers	- Experimental or quasi-experimental design—Yes, it is a comparative study. - Minimum duration—Yes, evaluation period with pre-post assessment.	This study compares different AI models rather than AI vs. no-AI.
Hany et al. (2024) [17]	Pre-post assessment design	- Experimental or quasi-experimental design—Yes, it is a pre-post design. - Minimum duration—Yes, ongoing tutoring.	The lack of a control group is a limitation of this study design.
Sukasih et al. (2024) [18]	Quasi-experimental design with pre-test and post-test	- Experimental or quasi-experimental design—Yes, it is a quasi-experimental design. - Minimum duration—Yes, 8 weeks.	The lack of a control group is a limitation of this study design.
Rizos et al. (2024) [11]	Case study with a comparison group	- Experimental or quasi-experimental design—Yes, it is a case study. - Minimum duration—Yes, 9 h.	The small sample size is a limitation of this study design.
Chukwuemeka & Agbarakwe (2024) [19]	Quasi-experimental design with three groups	- Experimental or quasi-experimental design—Yes, it is a quasi-experimental design. - Minimum duration—Yes, 3 weeks.	None. The study design is appropriate for the research question.
Samuelsson (2023) [7]	Randomized Controlled Trial (RCT) with four groups	- Experimental or quasi-experimental design—Yes, it is an RCT. - Minimum duration—Yes, 6 weeks.	None. The study design is appropriate and robust.
Fami et al. (2024) [20]	A-B-A single-subject research design	- Experimental or quasi-experimental design—Yes, it is a single-subject design. - Minimum duration—Yes, 10 sessions.	The small sample size is a limitation of this study design.

## Appendix B. Detailed Risk of Bias Assessment per Study

**Table A12:** Detailed Risk of Bias Assessment per Study (Part 1/2).

Study	Design	Tool Used
Zingoni et al. (2024) [12]	Descriptive/Case series	JBI Case Series
Ayasrah et al. (2024) [13]	Pre-post (single group)	JBI Quasi-Experimental
Morciano et al. (2024) [14]	Quasi-experimental	ROBINS-I
Gharaibeh et al. (2025) [15]	Quasi-experimental	ROBINS-I
Wang et al. (2022) [16]	Quasi-experimental	ROBINS-I
Hany et al. (2024) [17]	Descriptive/Case series	JBI Case Series
Sukasih et al. (2024) [18]	Quasi-experimental (pre-post)	JBI Quasi-Experimental
Rizos et al. (2024) [11]	Case study	JBI Case Series
Chukwuemeka & Agbarakwe (2024) [19]	Quasi-experimental	ROBINS-I
Samuelsson (2023) [7]	Quasi-experimental	ROBINS-I
Fami et al. (2024) [20]	Single-subject (A-B-A)	JBI Case Series

**Table A13:** Detailed Risk of Bias Assessment per Study (Part 2/2).

Study	Overall Risk	Main Notes
Zingoni et al. (2024) [12]	Moderate	Technological development study, limited preliminary testing
Ayasrah et al. (2024) [13]	High	Absence of control group, small sample (n = 15)
Morciano et al. (2024) [14]	Moderate	Algorithm development with controlled testing, lacks randomization
Gharaibeh et al. (2025) [15]	Moderate	Arabic dyslexia study, controlled design, blinded assessment
Wang et al. (2022) [16]	Serious	Technological development study, significant methodological limitations
Hany et al. (2024) [17]	High	Innovative AI system, insufficient preliminary evaluation
Sukasih et al. (2024) [18]	High	Well-structured mixed-methods, but absence of control group
Rizos et al. (2024) [11]	Moderate	Rigorous qualitative case study, innovative theoretical framework
Chukwuemeka & Agbarakwe (2024) [19]	Moderate	Three-arm controlled study, validated instruments, lacks randomization
Samuelsson (2023) [7]	Moderate	Large sample (n = 1006), high fidelity, non-randomized AI selection
Fami et al. (2024) [20]	Moderate	Rigorous pilot study, validated mixed program, appropriate A-B-A design
